# Maternal RANKL Reduces the Osteopetrotic Phenotype of Null Mutant Mouse Pups

**DOI:** 10.3390/jcm7110426

**Published:** 2018-11-08

**Authors:** Benjamin Navet, Jorge William Vargas-Franco, Andrea Gama, Jérome Amiaud, Yongwon Choi, Hideo Yagita, Christopher G. Mueller, Françoise Rédini, Dominique Heymann, Beatriz Castaneda, Frédéric Lézot

**Affiliations:** 1 INSERM, UMR 1238, Faculté de Médecine, Université de Nantes, F-44035 Nantes, France; Benjamin.navet@univ-nantes.fr (B.N.); jerome.amiaud@univ-nantes.fr (J.A.); Francoise.redini@univ-nantes.fr (F.R.); 2 Department of Basic Studies, Faculty of Odontology, University of Antioquia, Medellin AA 1226, Colombia; jorge.vargas@udea.edu.co; 3 INSERM, UMR-1138, Equipe 5, Centre de Recherche des Cordeliers, F-75006 Paris, France; dea.gama10@gmail.com (A.G.); Bea.castaneda.1@gmail.com (B.C.); 4 Department of Pathology and Laboratory Medicine, School of Medicine, University of Pennsylvania, Philadelphia, PA 19104, USA; ychoi3@pennmedicine.upenn.edu; 5 Department of Immunology, School of Medicine, Juntendo University, Tokyo 113-8421, Japan; hyagita@med.juntendo.ac.jp; 6 CNRS, UPR-9021, Laboratoire Immunologie et Chimie Thérapeutiques, Institut de Biologie Moléculaire et Cellulaire (IBMC), Université de Strasbourg, F-67084 Strasbourg, France; c.mueller@ibmc-cnrs.unistra.fr; 7 INSERM, LEA Sarcoma Research Unit, Department of Oncology and Human Metabolism, Medical School, University of Sheffield, Sheffield S10 2RX, UK; 8 INSERM, UMR 1232, LabCT, Université de Nantes, Université d’Angers, Institut de Cancérologie de l’Ouest, site René Gauducheau, F-44805 Saint-Herblain, France; Dominique.Heymann@univ-nantes.fr

**Keywords:** RANKL, skeletal growth, morphogenesis, osteoclast, bone, mandible, tooth

## Abstract

RANKL signalization is implicated in the morphogenesis of various organs, including the skeleton. Mice invalidated for *Rankl* present an osteopetrotic phenotype that was less severe than anticipated, depending on RANKL’s implication in morphogenesis. The hypothesis of an attenuated phenotype, as a result of compensation during gestation by RANKL of maternal origin, was thus brought into question. In order to answer this question, *Rankl* null mutant pups from null mutant parents were generated, and the phenotype analyzed. The results validated the presence of a more severe osteopetrotic phenotype in the second-generation null mutant with perinatal lethality. The experiments also confirmed that RANKL signalization plays a part in the morphogenesis of skeletal elements through its involvement in cell-to-cell communication, such as in control of osteoclast differentiation. To conclude, we have demonstrated that the phenotype associated with *Rankl* invalidation is attenuated through compensation by RANKL of maternal origin.

## 1. Introduction

RANKL (TNFSF11) signalization is implicated in the development, histogenesis, and functional homeostasis of various tissues, particularly lymphoid tissues, skin appendages (hair, teeth, and mammary glands) and skeletal components [1,2,3,4,5,6,7,8]. During development, expression of RANKL, as well as expressions of its receptors RANK and OPG, have been reported in the spleen [9], thymus [10,11], lymph nodes [12,13], hair [14], teeth [15,16], mammary glands [17,18], and bones, regardless of the ossification process involved: endochondral [19,20,21] or intramembranous [19,20]. In bone development, during endochondral ossification, RANKL expression by the pre-hypertrophic and hypertrophic chondrocytes is crucial for the differentiation and activation of osteoclasts that resorb the primary spongiosa, making trabecula formation possible [21,22,23]. During intramembranous ossification, RANKL is expressed by mesenchymal cells and actively synthesizes osteoblasts [19], which are of importance for woven bone resorption and replacement by lamellar bone. It was, therefore, unsurprising that loss of RANKL function was associated with osteoclast-poor osteopetrosis in young patients with RANKL mutations (autosomal recessive form, OPTB2; OMIM #259710; [24]) and in *Rankl* null mutant mice [1,5], as well as in monkeys or mice injected with a powerful RANKL-blocking antibody [25,26,27].

On the contrary, when RANKL was overexpressed, for instance, when produced genetically in pigs [28] and mice [29], a severe osteolytic phenotype was observed that led to premature death. This phenotype was in line with those associated with the gains in RANK function observed in patients with mutations (duplications) in the RANK signal peptide, leading to three seemingly homologous pathologies (familial expansile osteolysis, Paget disease of bone 2, expansile skeletal hyperphosphatasia) [30,31], as well as in mice overexpressing RANK [16,32].

All these observations underline the fact that finely tuned control of RANKL expression/function is required during the entire skeletal development process, from the early stages (antenatal) to adulthood.

RANKL was discovered as a cytoplasmic membrane-bound cytokine, but a soluble form was also evidenced [33]. Given the fact that RANKL is expressed in many tissues during embryonic development, and taking into account that soluble RANKL of maternal origin may cross the placenta, the question of the presence of an attenuated phenotype in young OPTB2 patients, as well as in *Rankl* null mutants from heterozygous mothers, was raised.

In order to answer this question, the skeletal phenotype of *Rankl* null mutant mice was compared at one day post-natal between mouse pups obtained from heterozygous vs. homozygous parents. Injections of a RANKL-blocking antibody were also performed on heterozygous mothers during the second half of gestation, to enhance the demonstration.

## 2. Materials and Methods

### 2.1. Animals and Drug Administration

All C57BL/6J mice used in the experiments were housed in pathogen-free conditions at the Experimental Therapy Unit at the medical faculty at the University of Nantes (Nantes, France), in accordance with the institutional guidelines of the French Ethical Committee (CEEA-PdL-06, accepted protocol number 00165.01) and under the supervision of authorized investigators. Newborn mice were used for the experiments.

The *Rankl* heterozygous mice were generated as previously described [5] by homologous recombination. Genotyping was carried out using PCR with the following primers 5′-*Rankl*: CCAAGTAGTGGATTCTAAATCCTG; 3′-*Rankl*: CCAACCTGTGGACTTACGATTAAAG; and 3′-insert: ATTCGCAGCGCATCGCCTTCTATC.

First- and second-generation null mutant pups were obtained by mating heterozygous and homozygous animals, respectively. The nomenclature used for the different animals obtained is presented in Supplementary Figure S1.

Some heterozygous pregnant mice were injected IP three times during the second part of gestation with 2 mg/kg of a mouse-specific RANKL-blocking antibody, IK22.5, following a protocol summarized in Supplementary Figure S1D.

### 2.2. MicroCT Analysis

Analyses of the bone microarchitecture were performed using a Skyscan 1076 in vivo microCT scanner (Skyscan, Kontich, Belgium). Tests were performed after sacrifice on the tibias and heads of each animal. All tibias and heads were scanned using the same parameters (pixel size 9 µm, 50 kV, 0.5 mm Al filter, 10 minutes of scanning). The reconstructions were analyzed using NRecon and CTan software (Skyscan, Kontich, Belgium). 3D visualizations of the tibias and heads were made using ANT software (Skyscan, Kontich, Belgium).

### 2.3. Histology

Whole skeletons, collected from euthanized one-day pups, were fixed in 4% buffered paraformaldehyde. Samples were decalcified in 4.13% EDTA/0.2% paraformaldehyde pH 7.4 over 4 days in KOS sw10 (Milestone, Sorisole, Italy). The specimens were dehydrated and embedded in paraffin. Then, 3 µm-thick sagittal sections stained with Masson’s trichrome were observed using a DMRXA microscope (Leica, Nussloch, Germany). Tartrate-resistant acid phosphatase (TRAP) staining was performed on sample sections to identify multinucleated osteoclast-like cells after 90 min’ incubation in 1 mg/mL of Naphthol AS-TR phosphate, 60 mmol/L *N*,*N*-dimethylformamide, 100 mmol/L sodium tartrate, and 1 mg/mL Fast Red TR Salt solution (all from Sigma Chemical Co., St. Louis, MO, USA), and counterstained with hematoxylin.

### 2.4. Immunohistochemistry

Immunohistochemistry was performed as previously described [34], using antibodies from Abcam (Cambridge, UK; ab75769 for CD146 and ab3697 for SOX9) and Bio-Rad (Marnes-la-Coquette, France; MCA1957 for CD68).

## 3. Results

### 3.1. Second-Generation Rankl Null Mutants Had a More Severe Craniofacial Phenotype

**Figure 1:**
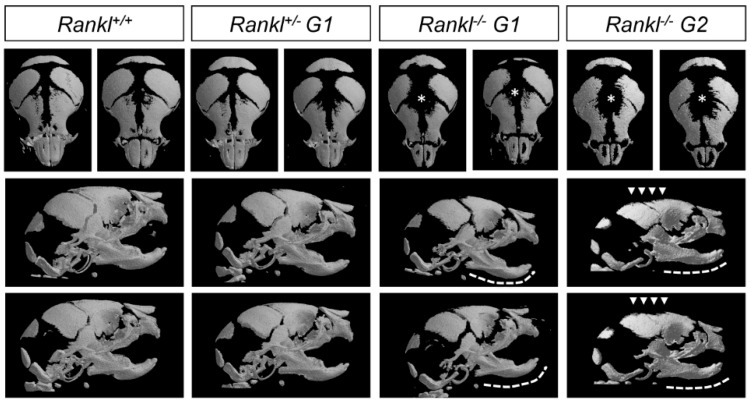
MicroCT comparative analysis of the craniofacial skeletons of first- and second-generation *Rankl* null mutant mice. Second-generation null mutants had enlarged foramen (stars and arrowheads) compared with first-generation null mutants. Percentage of closure measures (surface) for the different genotypes are 89 ± 2 for +/+, 84 ± 3 for +/−, 67 ± 4 for G1−/− and 57 ± 5 for G2−/−. Moreover, the mandibles of second-generation null mutants appeared flat, with missing proximo-distal curvature (dotted lines). Angle of the mandible curvature measures (opening degrees) for the different genotypes are 134 ± 9 for +/+, 136 ± 11 for +/−, 139 ± 9 for G1−/− and 163 ± 7 for G2−/−. No difference was observed between the wild type and heterozygous pups. Numbers of pups: 4 +/+, 8 G1+/−, 5 G1−/−, and 5 G2−/−.

**Figure 2:**
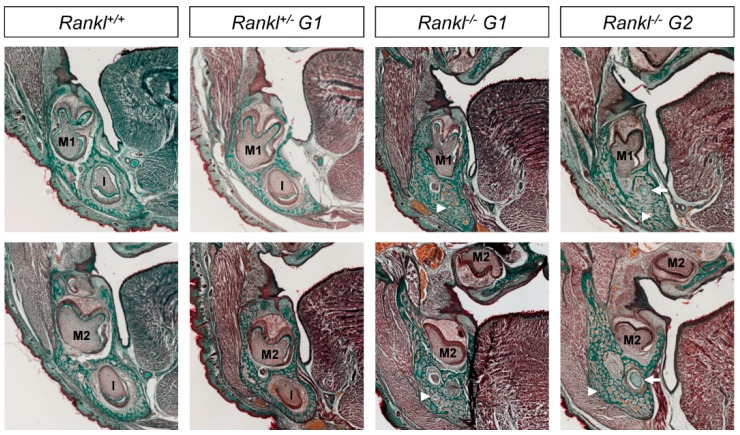
Histological comparative analysis of the craniofacial skeletons of first- and second-generation *Rankl* null mutant mice. Frontal sections of the head in planes of first (M1) and second (M2) molars revealed more severe osteopetrosis in the second-generation (*Rankl*^−^^/^^−^ *G2*) compared to the first-generation (*Rankl*^−^^/^^−^ *G1*) null mutants. While both null mutants revealed increased bone density compared to wild type and heterozygous mice (arrowheads), the second-generation null mutants had a remnant Meckel cartilage (arrow) and a very significant delay in the development of first and second molars, with the second molar appearing to be blocked between the cap and bell stages. Comparison of sections from wild type and heterozygous pups revealed a pre-osteopetrotic phenotype in the heterozygous mice, with increased bone density and delayed moving back of the incisor (I) in the mandible. Magnification 40× for all images. Numbers of pups: 4 +/+, 8 G1+/−, 5 G1−/−, and 5 G2−/−.

**Figure 3:**
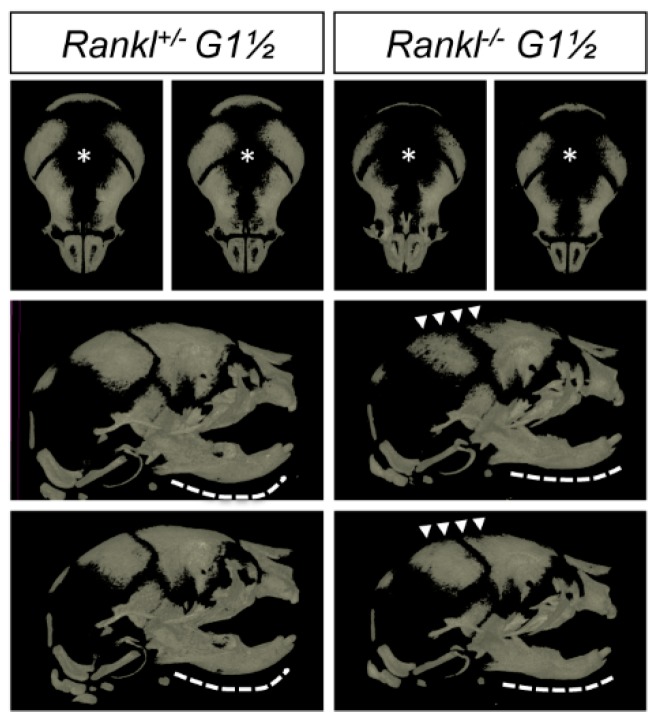
MicroCT comparative analysis of the craniofacial skeleton of pups born from null mutant females and heterozygous males. Heterozygous and null mutant pups revealed craniofacial phenotypes that were respectively similar to first- and second-generation null mutants. Enlarged foramina (stars) were present, more pronounced in the null mutants (arrowheads), and the mandibles of the null mutants appeared flat (dotted lines) as in the second-generation null mutants. Percentage of closure measures (surface) for the two genotypes are 65 ± 4 for +/− and 54 ± 7 for −/−. Angle of the mandible curvature measures (opening degrees) for the two genotypes are 133 ± 10 for +/−, 159 ± 12 for 1 −/−. Numbers of pups: 4 +/− and 2 −/−.

At birth, first-generation *Rankl* null mutants (*N* = 5) had a craniofacial osteopetrotic phenotype with an open foramen, due to delayed mineralization of the calvaria and delayed tooth development, associated with an absence of osteoclasts (Figure 1 and Figure 2). As part of the craniofacial skeleton develops during the second half of gestation, and taking into account that secreted forms of RANKL of maternal origin may be active in the embryo, the question of an attenuated craniofacial phenotype in first-generation *Rankl* null mutants was raised. In order to answer this question, second-generation *Rankl* null mutant pups were generated by mating male and female *Rankl* null mutants. The craniofacial phenotype of the second-generation null mutants (*N* = 5) was more severe than the phenotype of the first-generation mutants, with a more open foramen (Figure 1) and more delayed tooth morphogenesis (Figure 2). In addition, a loss in mandible curvature was observed (Figure 1), associated with defective disruption of the Meckel cartilage (Figure 2). A similar craniofacial phenotype was observed in null mutant pups (*N* = 2) from null mutant females mated with heterozygous males (Figure 3), confirming the importance of RANKL of maternal origin.

### 3.2. Second-Generation Rankl Null Mutants Had a More Severe Long Bone Phenotype

**Figure 4:**
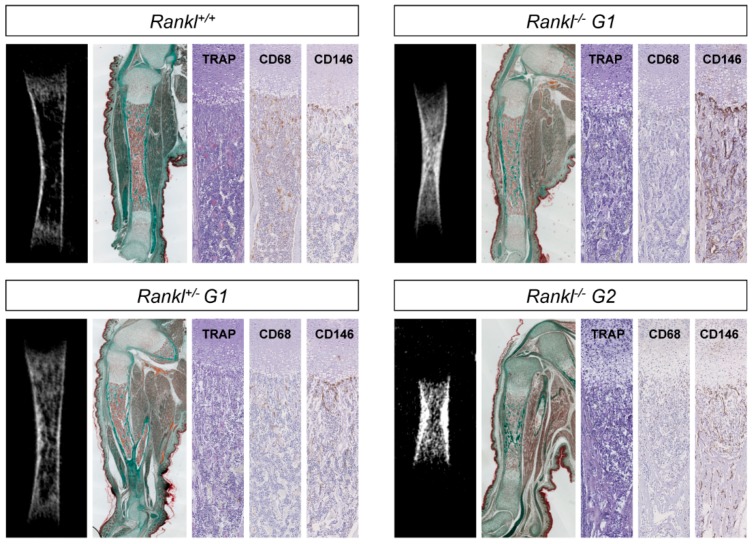
MicroCT (mCT) and histological comparative analysis of the appendicular skeleton of first- and second-generation *Rankl* null mutant mice. Tibias were chosen as representative bone of the appendicular skeleton. Second-generation null mutants revealed significantly delayed tibia development in comparison with first-generation null mutants, as shown by mCT and Masson trichrome staining. Interestingly, comparing the mCT and histological sections of wild type and heterozygous pups revealed a pre-osteopetrotic phenotype in the heterozygous pup. TRAP staining was observed in wild type and heterozygous pups, with a lower number of stained cells in the heterozygous pups. CD68 immunostaining decreased gradually (in intensity and number of stained-cells) from the wild type to the first-generation null mutant pups. No TRAP expression was observed in the second-generation null mutants, while small, round CD68 cells were visible in the subchondral area. CD146 immunostaining increased gradually (in terms of intensity and number of stained cells) from wild type to first-generation null mutant pups, while it was comparable in first- and second-generation null mutant pups. Masson trichrome magnification 10×; TRAP, CD68, and CD146 magnification 40×. Numbers of pups: 4 +/+, 8 G1+/−, 5 G1−/−, and 5 G2−/−.

**Figure 5:**
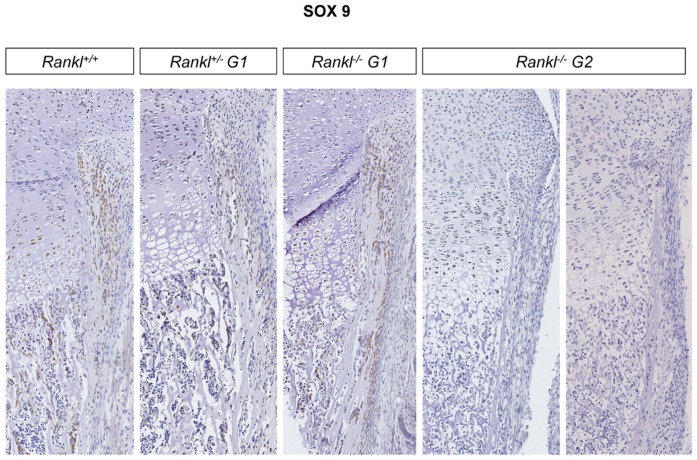
Immunohistochemical comparative analysis of SOX9 expression in the tibias of first- and second-generation *Rankl* null mutant mice. A graduated decrease in SOX9 expression was observed from wild type to first-generation null mutant pups in either chondroblastic cells or periosteal osteoblastic cells. In the tibias of second-generation null mutant pups, very weak staining was present in rare chondroblastic cells, while no staining was observed in periosteal osteoblastic cells. Magnification 100×. Numbers of pups: 4 +/+, 8 G1+/−, 5 G1−/−, and 5 G2−/−.

At birth, first-generation *Rankl* null mutants had a long bone osteopetrotic phenotype with delayed formation of the bone medullary cavity, as clearly seen by microtomography and histology (Figure 4). This appeared to be secondary to an absence of resorption of the spongy bone in the primary ossification center at the diaphysis site (TRAP staining in Figure 4). Interestingly, an intermediary phenotype was observed in the heterozygous mice, suggesting probable haplo-insufficiency (Figure 4). This was supported by graduated decreases in TRAP staining and CD68 monocyte/macrophage lineage immunodetection from the wild type through the heterozygous to the null mutant mice (Figure 4; Enlargements in Supplementary Figure S2). Moreover, a grade increase in detection of the vasculature marker (CD146) was revealed using immunohistochemistry from the WT through the heterozygous to the null mutant mice (Figure 4). The long bone phenotype of the second-generation null mutants was more severe than the phenotype of first-generation mutants (Figure 4; Enlargements in Supplementary Figure S2), suggesting an attenuated long bone phenotype in the first-generation null mutants. Interestingly, while no TRAP-positive cells were detected in the second-generation null mutant, small round CD68 cells seemed to accumulate in the restricted subchondral area (Figure 4), and there was significant CD146 staining. Surprisingly, immunohistochemistry revealed an almost complete lack of SOX9 expression, but only in the second-generation null mutants, with very weak staining in just a few chondroblastic cells, and no staining in the periosteal osteoblastic cells (Figure 5), whereas both mutants evidenced an apparently normal growth plate thickness with, however, disorganized chondrocyte columns (Figure 5).

### 3.3. IK22-5 RANKL-Blocking Antibody Injections in Pregnant Heterozygous Mice Induced a Second-Generation-Like Phenotype in Null Mutant Pups

**Figure 6:**
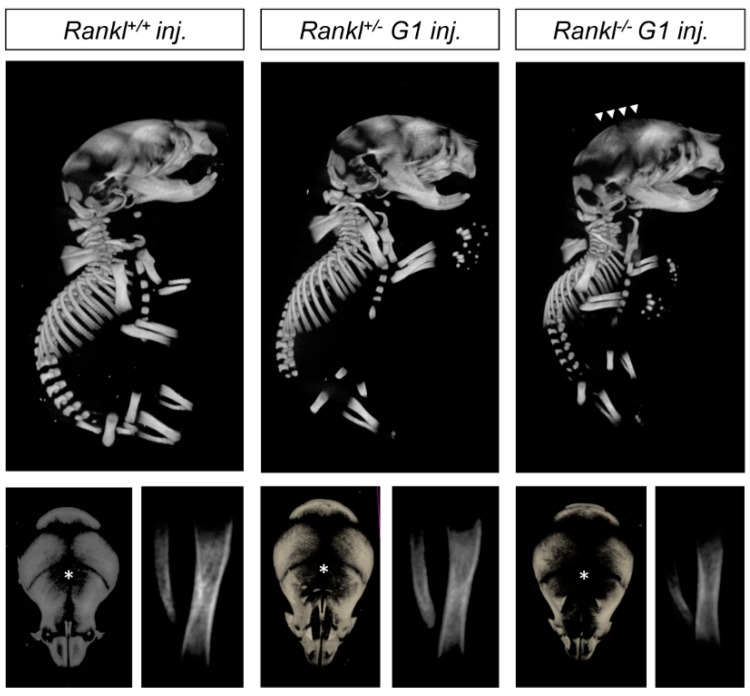
MicroCT comparative analysis of the skeletons of wild type, heterozygous, and first-generation *Rankl* null mutant pups born from females treated with IK22-5 during the second half of pregnancy. A graduated skeleton phenotype was observed from wild type to null mutant pups, with the presence of an enlarged foramen (star and arrowheads) in all genotypes. Percentage of closure measures (surface) for the different genotypes are 87 ± 3 for +/+, 68 ± 4 for +/−, and 60 ± 4 for −/−. Globally, it seems that injections of IK22-5 increased the phenotype of each genotype to the next in terms of severity, wild type being comparable to non-injected heterozygous, heterozygous to non-injected first-generation null mutants, and first-generation null mutants to second-generation phenotypes. Numbers of pups: 3 +/+, 6 +/−, and 2 −/−.

**Figure 7:**
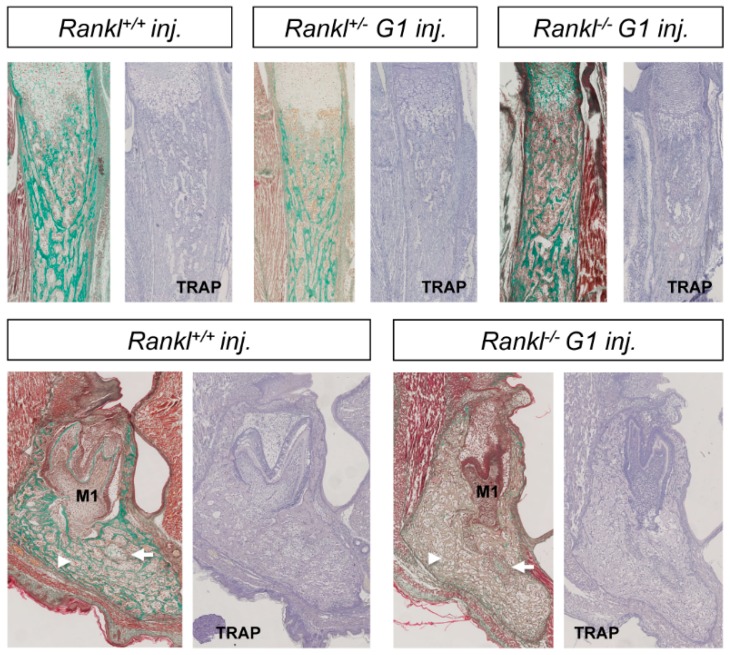
Histological comparative analysis of the skeletons of wild type, heterozygous, and first-generation *Rankl* null mutant pups born to females treated with IK22-5 during the second half of pregnancy. Masson trichrome staining of longitudinal sections of tibias made it possible to observe an osteopetrotic phenotype in all genotypes with, however, graduated severity from wild type to null mutant. TRAP staining was negative in all sections, signaling the absence of osteoclasts, induced by the IK22-5 injections. Masson trichrome staining of mandible frontal sections in the plane of the first molar (M1) revealed the induction of an osteopetrotic phenotype in the wild type pup with significant mandibular bone density (arrowhead), an absence of incisor in this section plane, remnant Meckel cartilage (arrow), and abnormal tooth morphology. TRAP staining of adjacent sections revealed a total absence of osteoclasts. Concerning the null mutant pups, a phenotype similar to the second-generation mutant pup was observed, with significant mandibular bone density (arrowhead), remnant Meckel cartilage (arrow), and abnormal tooth morphology. Magnification 40×. Numbers of pups: 3 +/+, 6 +/−, and 2 −/−.

In order to validate the importance of RANKL of maternal origin in the attenuated osteopetrotic phenotype of the null mutant pups, IK22-5-blocking antibody was injected into heterozygous pregnant females during the second part of gestation, and the consequences on the whole skeleton of the pups were analyzed (Figure 6 and Figure 7). A noticeable aggravation in either craniofacial or long bone phenotypes was observed for the different genotypes, with WT being comparable to first-generation heterozygous mutants and first-generation to second-generation null mutants. Surprisingly, while a more open foramen (Figure 6) and severe tooth morphogenesis delay (Figure 7) was observed in the null mutants from injected mothers, the curvature of the mandible appeared unaffected (Figure 6), suggesting that this craniofacial osteopetrotic feature was secondary to a loss of RANKL function before mid-gestation.

## 4. Discussion

During embryonic development, RANKL expression has been reported in several tissues, with suggested implications in the cell-to-cell communications necessary for the morphogenesis of the corresponding organs, such as teeth, bones, thymus, thyroid glands, and lymph nodes. Surprisingly, RANKL invalidation in mice did not induce premature lethality, despite a severe osteopetrotic phenotype and alterations to the immune system (for reviews, see [8,35]). Moreover, the *Rankl* null mutant osteopetrotic phenotype appears less severe than that reported for its main receptor, *Rank* null mutant [36]. Depending on the existence of soluble forms of RANKL that may cross the placenta, the question of an attenuated phenotype in the null mutant due to partial compensation by RANKL of maternal origin was raised. In order to answer this question, second-generation null mutants were generated, and the osteopetrotic phenotype compared with first-generation mutants. The data obtained validated the existence of partial compensation of this type in the entire skeleton, suggesting that soluble RANKL of maternal origin does, indeed, cross the placenta.

Second-generation null mutants were difficult to obtain because of considerable embryonic lethality, as also reported by another group and explained by defects in decidual M2 macrophage polarization, essential for maternal-fetal tolerance [37]. The mutants that survived until birth did not survive more than 30 h, even if adopted by wild type lactating females (to deal with the fact that mammary gland development was defective in the null mutant mother). These observations validated the notion that normal embryonic development requires RANKL. Considering the fact that early lethality was also reported in transgenic pigs and mice following overexpression of RANKL [28,29], it appeared that, during embryonic development, the RANKL expression level required a strict regulation.

Blood concentrations of RANKL and its decoy receptor OPG were analyzed throughout normal and pathological pregnancies, supporting the role played by RANKL signalization in communications between the mother and the fetus [38,39,40,41,42,43]. Alterations to RANKL and OPG expressions were associated with severe gestational pathologies, such as pre-eclampsia [38,40,41], intrauterine growth restriction [39,42], and premature labor [43], negatively impacting the life expectancy of both the mother and infant. Further studies are needed to decipher the complex role played by RANKL signalization during gestation, bearing in mind that RANKL has at least three receptors, two at the membrane of target cells, RANK and LGR4 [44], and one secreted, OPG.

Based on the data presented concerning skeletal development, RANKL signalization appears to have two main functions: controlling osteoclast differentiation, and in communication between mineral tissues forming cells. It has consequences on morphogenesis and histogenesis.

For the first function, in long bones such as the tibia, the total absence of osteoclasts was responsible for both a significant delay in development, and an absence of bone marrow space formation. The initial vascularization appeared to be maintained, and an accumulation of potential osteoclast precursors, CD68 cells, could be observed around the vessels. Moreover, the expression of SOX9, a major factor in endochondral bone formation [45], was severely decreased in the second-generation null mutants, outlining that the absence of osteoclasts completely blocked long bone formation. With regard to the craniofacial skeleton, the absence of osteoclasts made resorption of the Meckel cartilage impossible, which is an important step in mandible growth (for a review, see [46]).

The significant delay in tooth morphogenesis, observed in the second-generation null mutants, validated the considerable involvement of RANKL signalization in the communication between mineral tissues forming cells, which is the second function of RANKL signalization during skeletal development. The existence of such a function in teeth was initially suggested by the expression patterns of elements of RANKL signalization during morphogenesis [15]. Further studies are needed to decipher the modalities of this type of function, and the ability of the RANKL-blocking antibody to cross the placenta will be helpful. The absence of mandible curvature observed in the second-generation null mutants was also a consequence of the loss of the cell-to-cell communication function of RANKL signalization. Similar flat mandibles were reported in mice invalidated for transcription factors known to have morphogenetic functions, such as MSX1 [47] and PAX9 [48]. Crosstalk between RANKL signalization and these transcription factors may exist, as previously reported for other transcription factors, namely MSX2, EN1, and DLXs [32,49,50]. Further studies are needed to demonstrate the veracity of such crosstalk and its implications in morphogenesis of the whole skeleton.

## 5. Conclusions

To conclude, second-generation *Rankl* null mutants made it possible to demonstrate that the pediatric osteopetrotic phenotype associated with loss of RANKL function was reduced, thanks to partial compensation by RANKL of maternal origin during gestation. Those animals also made it possible to validate the involvement of RANKL signalization functionally in the communications between the mother and the embryo, but also in the morphogenesis/histogenesis of different organs through its implication in cell-to-cell communication and osteoclast differentiation control.

## Supplementary Materials

The following are available online at http://www.mdpi.com/2077-0383/7/11/426/s1, Figure S1, Nomenclature used to distinguish the different mutants based on the genotypes of the parents, both heterozygous (A), both homozygous (B), and only the mother homozygous (C). The chronogram used for IK22-5 injections in pregnant heterozygous mice is also presented (D); Figure S2, Enlargements of Figure 4 and Figure 7 enabling to visualize the different tibia growth plate cartilage phenotypes at the cellular level. Magnification 100×.

## References

[1] Kong Y.Y., Yoshida H., Sarosi I., Tan H.L., Timms E., Capparelli C., Morony S., Oliveira-dos-Santos A.J., Van G., Itie A. (1999). OPGL is a key regulator of osteoclastogenesis, lymphocyte development and lymph-node organogenesis. Nature.

[2] Kong Y.Y., Boyle W.J., Penninger J.M. (1999). Osteoprotegerin ligand: A common link between osteoclastogenesis, lymph node formation and lymphocyte development. Immunol. Cell Biol..

[3] Fata J.E., Kong Y.Y., Li J., Sasaki T., Irie-Sasaki J., Moorehead R.A., Elliott R., Scully S., Voura E.B., Lacey D.L. (2000). The osteoclast differentiation factor osteoprotegerin-ligand is essential for mammary gland development. Cell.

[4] Kim D., Mebius R.E., MacMicking J.D., Jung S., Cupedo T., Castellanos Y., Rho J., Wong B.R., Josien R., Kim N. (2000). Regulation of peripheral lymph node genesis by the tumor necrosis factor family member TRANCE. J. Exp. Med..

[5] Kim N., Odgren P.R., Kim D.K., Marks S.C., Choi Y. (2000). Diverse roles of the tumor necrosis factor family member TRANCE in skeletal physiology revealed by TRANCE deficiency and partial rescue by a lymphocyte-expressed TRANCE transgene. Proc. Natl. Acad. Sci. USA.

[6] Xing L., Schwarz E.M., Boyce B.F. (2005). Osteoclast precursors, RANKL/RANK, and immunology. Immunol. Rev..

[7] Sugiyama M., Nakato G., Jinnohara T., Akiba H., Okumura K., Ohno H., Yoshida H. (2012). Expression pattern changes and function of RANKL during mouse lymph node microarchitecture development. Int. Immunol..

[8] Walsh M.C., Choi Y. (2014). Biology of the RANKL-RANK-OPG System in Immunity, Bone, and Beyond. Front. Immunol..

[9] Habbeddine M., Verthuy C., Rastoin O., Chasson L., Bebien M., Bajenoff M., Adriouch S., den Haan J.M.M., Penninger J.M., Lawrence T. (2017). Receptor Activator of NF-κB Orchestrates Activation of Antiviral Memory CD8 T Cells in the Spleen Marginal Zone. Cell Rep..

[10] Hikosaka Y., Nitta T., Ohigashi I., Yano K., Ishimaru N., Hayashi Y., Matsumoto M., Matsuo K., Penninger J.M., Takayanagi H. (2008). The cytokine RANKL produced by positively selected thymocytes fosters medullary thymic epithelial cells that express autoimmune regulator. Immunity.

[11] Desanti G.E., Cowan J.E., Baik S., Parnell S.M., White A.J., Penninger J.M., Lane P.J.L., Jenkinson E.J., Jenkinson W.E., Anderson G. (2012). Developmentally regulated availability of RANKL and CD40 ligand reveals distinct mechanisms of fetal and adult cross-talk in the thymus medulla. J. Immunol..

[12] Mueller C.G., Hess E. (2012). Emerging Functions of RANKL in Lymphoid Tissues. Front. Immunol..

[13] Hess E., Duheron V., Decossas M., Lézot F., Berdal A., Chea S., Golub R., Bosisio M.R., Bridal S.L., Choi Y. (2012). RANKL Induces Organized Lymph Node Growth by Stromal Cell Proliferation. J. Immunol..

[14] Duheron V., Hess E., Duval M., Decossas M., Castaneda B., Klöpper J.E., Amoasii L., Barbaroux J.-B., Williams I.R., Yagita H. (2011). Receptor activator of NF-kappaB (RANK) stimulates the proliferation of epithelial cells of the epidermo-pilosebaceous unit. Proc. Natl. Acad. Sci. USA.

[15] Ohazama A., Courtney J.-M., Sharpe P.T. (2004). Opg, Rank, and Rankl in tooth development: Co-ordination of odontogenesis and osteogenesis. J. Dent. Res..

[16] Castaneda B., Simon Y., Jacques J., Hess E., Choi Y.-W., Blin-Wakkach C., Mueller C., Berdal A., Lézot F. (2011). Bone resorption control of tooth eruption and root morphogenesis: Involvement of the receptor activator of NF-κB (RANK). J. Cell. Physiol..

[17] Kim N.-S., Kim H.-J., Koo B.-K., Kwon M.-C., Kim Y.-W., Cho Y., Yokota Y., Penninger J.M., Kong Y.-Y. (2006). Receptor activator of NF-kappaB ligand regulates the proliferation of mammary epithelial cells via Id2. Mol. Cell. Biol..

[18] Tanos T., Brisken C. (2008). What signals operate in the mammary niche?. Breast Dis..

[19] Kartsogiannis V., Zhou H., Horwood N.J., Thomas R.J., Hards D.K., Quinn J.M., Niforas P., Ng K.W., Martin T.J., Gillespie M.T. (1999). Localization of RANKL (receptor activator of NF kappa B ligand) mRNA and protein in skeletal and extraskeletal tissues. Bone.

[20] Sakakura Y., Tsuruga E., Irie K., Hosokawa Y., Nakamura H., Yajima T. (2005). Immunolocalization of receptor activator of nuclear factor-kappaB ligand (RANKL) and osteoprotegerin (OPG) in Meckel’s cartilage compared with developing endochondral bones in mice. J. Anat..

[21] Xiong J., Onal M., Jilka R.L., Weinstein R.S., Manolagas S.C., O’Brien C.A. (2011). Matrix-embedded cells control osteoclast formation. Nat. Med..

[22] Odgren P.R., Witwicka H., Reyes-Gutierrez P. (2016). The cast of clasts: Catabolism and vascular invasion during bone growth, repair, and disease by osteoclasts, chondroclasts, and septoclasts. Connect. Tissue Res..

[23] Atkins G.J., Kostakis P., Pan B., Farrugia A., Gronthos S., Evdokiou A., Harrison K., Findlay D.M., Zannettino A.C.W. (2003). RANKL expression is related to the differentiation state of human osteoblasts. J. Bone Miner. Res..

[24] Sobacchi C., Frattini A., Guerrini M.M., Abinun M., Pangrazio A., Susani L., Bredius R., Mancini G., Cant A., Bishop N. (2007). Osteoclast-poor human osteopetrosis due to mutations in the gene encoding RANKL. Nat. Genet..

[25] Boyce R.W., Varela A., Chouinard L., Bussiere J.L., Chellman G.J., Ominsky M.S., Pyrah I.T. (2014). Infant cynomolgus monkeys exposed to denosumab in utero exhibit an osteoclast-poor osteopetrotic-like skeletal phenotype at birth and in the early postnatal period. Bone.

[26] Lézot F., Chesneau J., Navet B., Gobin B., Amiaud J., Choi Y., Yagita H., Castaneda B., Berdal A., Mueller C.G. (2015). Skeletal consequences of RANKL-blocking antibody (IK22-5) injections during growth: Mouse strain disparities and synergic effect with zoledronic acid. Bone.

[27] Okamatsu N., Sakai N., Karakawa A., Kouyama N., Sato Y., Inagaki K., Kiuchi Y., Oguchi K., Negishi-Koga T., Takami M. (2017). Biological effects of anti-RANKL antibody administration in pregnant mice and their newborns. Biochem. Biophys. Res. Commun..

[28] Klymiuk N., Böcker W., Schönitzer V., Bähr A., Radic T., Fröhlich T., Wünsch A., Keßler B., Kurome M., Schilling E. (2012). First inducible transgene expression in porcine large animal models. FASEB J..

[29] Mizuno A., Kanno T., Hoshi M., Shibata O., Yano K., Fujise N., Kinosaki M., Yamaguchi K., Tsuda E., Murakami A. (2002). Transgenic mice overexpressing soluble osteoclast differentiation factor (sODF) exhibit severe osteoporosis. J. Bone Miner. Metab..

[30] Hughes A.E., Ralston S.H., Marken J., Bell C., MacPherson H., Wallace R.G., van Hul W., Whyte M.P., Nakatsuka K., Hovy L. (2000). Mutations in TNFRSF11A, affecting the signal peptide of RANK, cause familial expansile osteolysis. Nat. Genet..

[31] Palenzuela L., Vives-Bauza C., Fernández-Cadenas I., Meseguer A., Font N., Sarret E., Schwartz S., Andreu A.L. (2002). Familial expansile osteolysis in a large Spanish kindred resulting from an insertion mutation in the TNFRSF11A gene. J. Med. Genet..

[32] Castaneda B., Simon Y., Ferbus D., Robert B., Chesneau J., Mueller C., Berdal A., Lézot F. (2013). Role of RANKL (TNFSF11)-dependent osteopetrosis in the dental phenotype of Msx2 null mutant mice. PLoS ONE.

[33] Ikeda T., Kasai M., Utsuyama M., Hirokawa K. (2001). Determination of three isoforms of the receptor activator of nuclear factor-kappaB ligand and their differential expression in bone and thymus. Endocrinology.

[34] Sojod B., Chateau D., Mueller C.G., Babajko S., Berdal A., Lézot F., Castaneda B. (2017). RANK/RANKL/OPG Signalization Implication in Periodontitis: New Evidence from a RANK Transgenic Mouse Model. Front. Physiol..

[35] Leibbrandt A., Penninger J.M. (2008). RANK/RANKL: Regulators of immune responses and bone physiology. Ann. N. Y. Acad. Sci..

[36] Xing L., Chen D., Boyce B.F. (2013). Mice Deficient in NF-κB p50 and p52 or RANK Have Defective Growth Plate Formation and Post-natal Dwarfism. Bone Res..

[37] Meng Y.-H., Zhou W.-J., Jin L.-P., Liu L.-B., Chang K.-K., Mei J., Li H., Wang J., Li D.-J., Li M.-Q. (2017). RANKL-mediated harmonious dialogue between fetus and mother guarantees smooth gestation by inducing decidual M2 macrophage polarization. Cell Death Dis..

[38] Shaarawy M., Zaki S., Ramzi A.-M., Salem M.E., El-Minawi A.M. (2005). Feto-maternal bone remodeling in normal pregnancy and preeclampsia. J. Soc. Gynecol. Investig..

[39] Briana D.D., Boutsikou M., Baka S., Hassiakos D., Gourgiotis D., Malamitsi-Puchner A. (2009). Circulating osteoprotegerin and sRANKL concentrations in the perinatal period at term. The impact of intrauterine growth restriction. Neonatology.

[40] Vitoratos N., Lambrinoudaki I., Rizos D., Armeni E., Alexandrou A., Creatsas G. (2011). Maternal circulating osteoprotegerin and soluble RANKL in pre-eclamptic women. Eur. J. Obstet. Gynecol. Reprod. Biol..

[41] Shen P., Gong Y., Wang T., Chen Y., Jia J., Ni S., Zhou B., Song Y., Zhang L., Zhou R. (2012). Expression of osteoprotegerin in placenta and its association with preeclampsia. PLoS ONE.

[42] Tenta R., Bourgiezi I., Aliferis E., Papadopoulou M., Gounaris A., Skouroliakou M. (2013). Bone metabolism compensates for the delayed growth in small for gestational age neonates. Organogenesis.

[43] Rzepka R., Dołęgowska B., Sałata D., Rajewska A., Budkowska M., Domański L., Kwiatkowski S., Mikołajek-Bedner W., Torbé A. (2015). Soluble receptors for advanced glycation end products and receptor activator of NF-κB ligand serum levels as markers of premature labor. BMC Pregnancy Childbirth.

[44] Luo J., Yang Z., Ma Y., Yue Z., Lin H., Qu G., Huang J., Dai W., Li C., Zheng C. (2016). LGR4 is a receptor for RANKL and negatively regulates osteoclast differentiation and bone resorption. Nat. Med..

[45] Hattori T., Müller C., Gebhard S., Bauer E., Pausch F., Schlund B., Bösl M.R., Hess A., Surmann-Schmitt C., von der Mark H. (2010). SOX9 is a major negative regulator of cartilage vascularization, bone marrow formation and endochondral ossification. Dev. Camb. Engl..

[46] Parada C., Chai Y. (2015). Mandible and Tongue Development. Curr. Top. Dev. Biol..

[47] Orestes-Cardoso S., Nefussi J.R., Lezot F., Oboeuf M., Pereira M., Mesbah M., Robert B., Berdal A. (2002). Msx1 is a regulator of bone formation during development and postnatal growth: In vivo investigations in a transgenic mouse model. Connect. Tissue Res..

[48] Anthwal N., Peters H., Tucker A.S. (2015). Species-specific modifications of mandible shape reveal independent mechanisms for growth and initiation of the coronoid. EvoDevo.

[49] Lézot F., Thomas B.L., Blin-Wakkach C., Castaneda B., Bolanos A., Hotton D., Sharpe P.T., Heymann D., Carles G.F., Grigoriadis A.E. (2010). Dlx homeobox gene family expression in osteoclasts. J. Cell. Physiol..

[50] Deckelbaum R.A., Majithia A., Booker T., Henderson J.E., Loomis C.A. (2006). The homeoprotein engrailed 1 has pleiotropic functions in calvarial intramembranous bone formation and remodeling. Dev. Camb. Engl..

