# Supplementary material for: Maternal RANKL Reduces the Osteopetrotic Phenotype of Null Mutant Mouse Pups
Source: J Clin Med. 2018 Nov 8;7(11):426. doi: 10.3390/jcm7110426 (PMC6262436; doi:10.3390/jcm7110426)
Supplement: Supplementary file 1 [file jcm-07-00426-s001.zip › jcm-379029-Supplementary Figure S1.pdf]

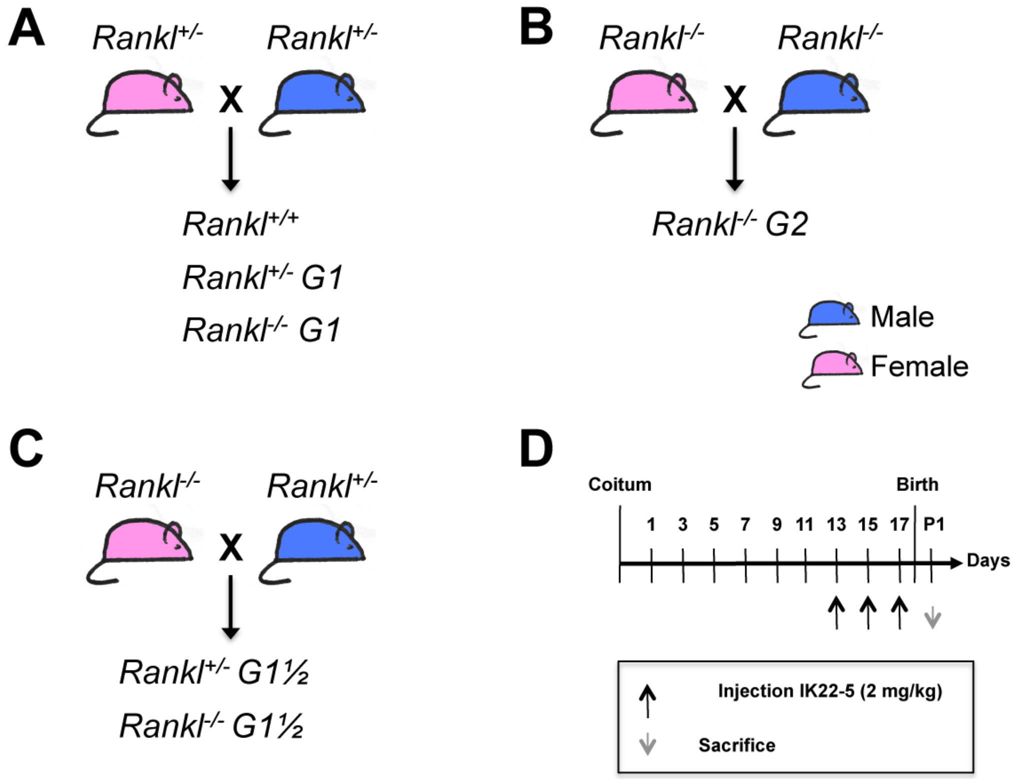

Supplemental Figure 1: Nomenclature used to distinguish the different mutants based on the genotypes of the parents, both heterozygous (A), both homozygous (B) and only the mother homozygous (C). The chronogram used for IK22-5 injections in pregnant heterozygous mice is also presented (D).
