# Supplementary material for: Maternal RANKL Reduces the Osteopetrotic Phenotype of Null Mutant Mouse Pups
Source: J Clin Med. 2018 Nov 8;7(11):426. doi: 10.3390/jcm7110426 (PMC6262436; doi:10.3390/jcm7110426)
Supplement: Supplementary file 1 [file jcm-07-00426-s001.zip › jcm-379029-Supplementary Figure S2.pdf]

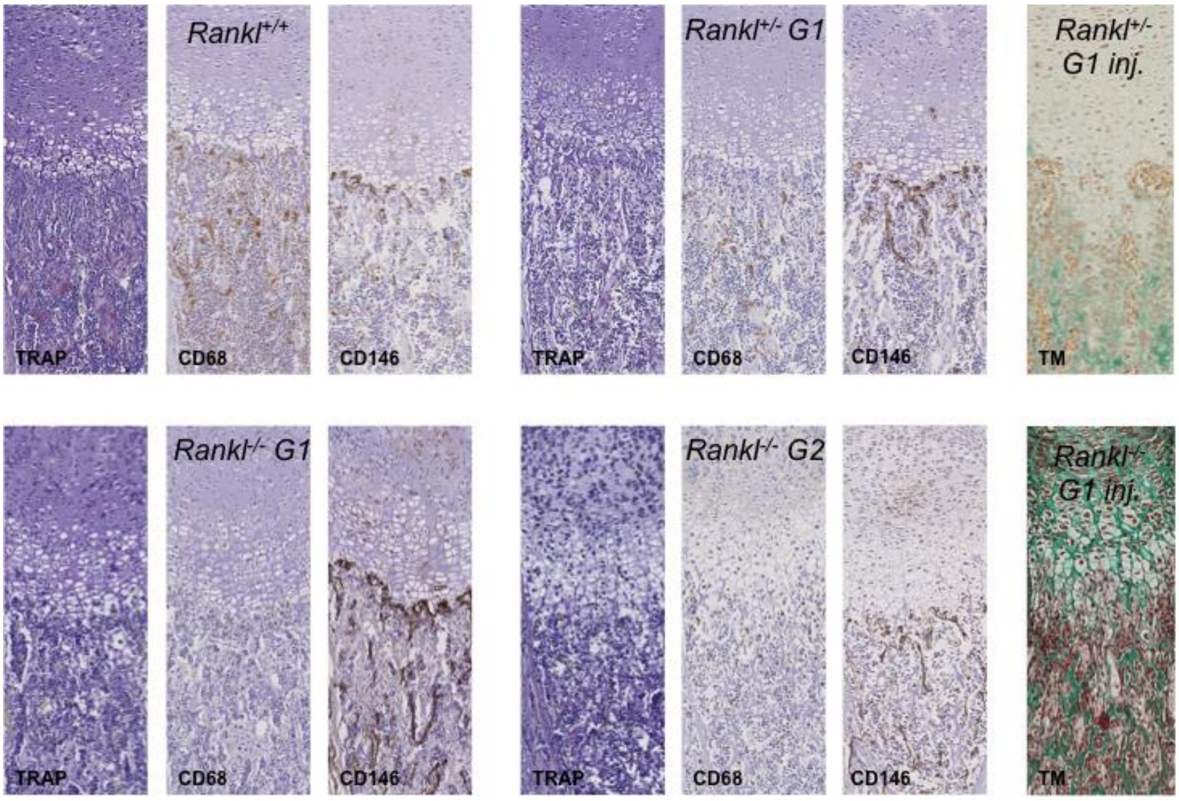

Supplementary Figure S2. Enlargements of Figures 4 and 7 enabling to visualize the different tibia growth plate cartilage phenotypes at the cellular level. Magnification X100.
